# Evaluating the Effectiveness of Self-Administration of Medication (SAM) Schemes in the Hospital Setting: A Systematic Review of the Literature

**DOI:** 10.1371/journal.pone.0113912

**Published:** 2014-12-02

**Authors:** Suzanna J. Richardson, Hannah L. Brooks, George Bramley, Jamie J. Coleman

**Affiliations:** 1 University Hospitals Birmingham NHS Foundation Trust, Edgbaston, Birmingham, B15 2WB, United Kingdom; 2 Primary Care Clinical Sciences, School of Health & Population Sciences, University of Birmingham, Birmingham, B15 2TT, United Kingdom; 3 School of Clinical and Experimental Medicine, College of Medical and Dental Sciences, University of Birmingham, Birmingham B15 2TT, United Kingdom; Stanford University School of Medicine, United States of America

## Abstract

**Background:**

Self-administration of medicines is believed to increase patients' understanding about their medication and to promote their independence and autonomy in the hospital setting. The effect of inpatient self-administration of medication (SAM) schemes on patients, staff and institutions is currently unclear.

**Objective:**

To systematically review the literature relating to the effect of SAM schemes on the following outcomes: patient knowledge, patient compliance/medication errors, success in self-administration, patient satisfaction, staff satisfaction, staff workload, and costs.

**Design:**

Keyword and text word searches of online databases were performed between January and March 2013. Included articles described and evaluated inpatient SAM schemes. Case studies and anecdotal studies were excluded.

**Results:**

43 papers were included for final analysis. Due to the heterogeneity of results and unclear findings it was not possible to perform a quantitative synthesis of results. Participation in SAM schemes often led to increased knowledge about drugs and drug regimens, but not side effects. However, the effect of SAM schemes on patient compliance/medication errors was inconclusive. Patients and staff were highly satisfied with their involvement in SAM schemes.

**Conclusions:**

SAM schemes appear to provide some benefits (e.g. increased patient knowledge), but their effect on other outcomes (e.g. compliance) is unclear. Few studies of high methodological quality using validated outcome measures exist. Inconsistencies in both measuring and reporting outcomes across studies make it challenging to compare results and draw substantive conclusions about the effectiveness of SAM schemes.

## Introduction

The Royal Pharmaceutical Society (RPS) describes patient self-administration of medication (SAM) as a ‘transfer of responsibility’ which should be dependent on a patient's ability to manage the tasks involved, as well as giving their consent to do so [1]. The concept of inpatient SAM has been around for more than 60 years [2] and self-administration is encouraged in many hospitals worldwide [3]–[6]. The National Prescribing Centre (NPC) in the UK describes the aims of SAM as establishing ‘a standardised approach for determining the ability of patients to take their own medication correctly and safely, increase the patient's knowledge and understanding of their medication, and promote and maintain patient independence and autonomy' [7].

Hospital SAM schemes often incorporate several stages of increasing patient independence and decreasing nurse involvement as patients become more competent [7]. Within different SAM schemes this transition to independence often includes different numbers of stages and variations in the roles of patients and nurses within each stage. For example, nurses may initially administer medications to patients. Following this, patients may request medications from the nurse and self-administer their medications under observation. Eventually, patients may become completely responsible for administering their medication regimen, with nurses or pharmacists intermittently checking that patients are administering their medications correctly using, for example, random pill counts or covert observations.

For many patients, it is important to be able to self-administer their medications successfully as they will often be expected to do so once they are discharged from hospital. In line with this, the UK's National Institute for Health and Care Excellence (NICE) states that prescribers should, ‘when possible, support patients to take responsibility for their medicines and self-manage their conditions' [8]. However, the NPC emphasises that allowing patients to self-administer, ‘…must balance the benefits to patients against the risks introduced’ [7].

It has been proposed that patients, staff and healthcare systems will benefit from the implementation of a SAM scheme [9], [10]; however the evidence is not clear cut. Two previously published English language literature reviews on the effectiveness of SAM schemes [11], [12] both concluded that there is a dearth of well-designed studies, with a lack of validated measures and inadequate reporting strategies. Therefore, it is difficult to fully understand whether SAM schemes are beneficial to patients and/or institutions that implement them. No active systematic reviews were found following a thorough search of databases. Therefore, in order to assess the effectiveness of SAM schemes for improving patient, staff and system outcomes and to reassess the evidence from previous publications, we performed a systematic review of the literature to evaluate the effectiveness of implementing SAM schemes in hospitals.

### Study objective

We reviewed the literature in order to answer the following questions about hospital inpatient self-administration:

1. What types of SAM scheme interventions have been implemented by healthcare professionals among hospital inpatients?
2. What effects have these interventions had on outcomes related to patients, staff and institutions?

## Methods

We undertook a systematic search of the literature and a narrative synthesis. Full details of the study protocol can be accessed via PROSPERO [reference CRD42013003498] [13].

### Criteria for considering studies for this review

#### Types of studies

We included randomised controlled trials (RCTs), non-RCTs, cohort studies, observational studies, cross-sectional studies, before-and-after studies, and case series. Case studies and anecdotal studies were excluded, as were those which did not evaluate the scheme or provided insufficient information about the evaluation.

#### Types of participants

We included studies that reported evaluations of SAM schemes implemented in a hospital inpatient setting. We excluded texts for the following reasons: where patients were assisted by relatives or carers, as this may negate the experience of self-administering; where drugs were administered as needed by patients (PRN), as these did not follow a pre-specified regimen; studies which described outpatient or community-based interventions; or where patients would use their own (prescribed or otherwise) drugs that were not dispensed by the hospital. The reasons for excluding the latter group were that patients' own drugs are generally considered to reduce medication waste and aid the reconciliation process on admission, and is not necessarily an indicator of self-administration. In addition, we felt the use of the patient's own drugs for self-administration may be more likely to have been initiated by the patient, as opposed to a structured scheme implemented by the healthcare team, as our objective states.

#### Types of outcome measures

We included studies that provided information on at least one of the following primary outcome measures: patient knowledge and patient compliance or error. We also investigated a number of secondary outcome measures: patient success in self-administering; patient satisfaction; staff satisfaction; staff workload; and costs.

### Search strategy

We performed a comprehensive search (see Table S1) based on a combination of text words and controlled vocabulary search terms using the following databases: EMBASE via OVID; MEDLINE via OVID; Cumulative Index to Nursing and Allied Health Literature (CINAHL) Plus via OVID; PsycINFO via OVID; Health Management Information Consortium (HMIC) via OVID; National Electronic Library for Medicines (NELM; previously PharmLine); Conference Proceedings Citation Index – Science (CPCI-S) and Conference Proceedings Citation Index – Social Science and Humanities (CPCI- SSH) via Web of Knowledge; Zetoc; Clinical Trials Register; World Health Organization's Clinical Trials Registry Platform; National Institute for Health Research Health Technology Assessments (NIHR HTA). Prior to our main search we checked the following databases for recent or ongoing similar reviews: PROPSPERO, Database of Abstracts of Reviews of Effects (DARE), Cochrane Database of Systematic Reviews (CDSR), Campbell Collaboration & Evidence for Policy and Practice Information's Database of Promoting Health Effectiveness Reviews (EPPI: DoPHER). Some of the databases (CINAHL, CPCI-S, CPCI-SSH, HMIC, and Zetoc) contained grey literature, which we chose to include in our review if it met the inclusion and exclusion criteria. Key search terms included: self-administration; hospital; compliance; error; patient satisfaction; knowledge; self-efficacy; drug; and medication (see Table S1 for full search strategy). The searches were performed between January and March 2013, with searches including studies from between 1950–1993 (each database offered a different search data range) to March 2013. In addition, we identified additional relevant articles cited in the selected texts. Searches were not limited by language or publication status.

### Data collection and analysis

#### Selection of studies

Titles and abstracts of papers identified in the search were reviewed independently by two of four review authors (SR, HB, GB or JC), and articles that appeared to fulfil the inclusion criteria were retrieved. The degree of agreement for each pair of reviewers was calculated using Kappa coefficients. Where there were discrepancies, a decision to include or exclude the study was reached by consensus between the two reviewers, or if necessary a third reviewer was also involved. From the full text of these papers, three review authors (SR, HB, GB) independently established whether studies met the inclusion criteria.

Included texts were assessed for quality of reporting using a modified version of the quality assessment checklist for health care interventions developed by Downs and Black [14]. In the original checklist, the final question (number 27) regarding statistical power is awarded between 0 and 5 points. We found, like other studies [15]–[17] the scoring criteria to be unclear and so limited our score to either 0 (study includes no discussion of statistical power) or 1 (study includes discussion of statistical power). This scoring method was also used by Deshpande et al [14]. The maximum possible score on the checklist was 28 points, with a higher score indicating a higher quality study.

Studies were assessed independently by two of three reviewers (SR, HB or GB) and the third reviewer acted as adjudicator where consensus could not be reached.

#### Data extraction and analysis

For each text, two of the three aforementioned reviewers independently undertook data extraction using a piloted data extraction form. Information was collated on rationale, aims and design of intervention, population, country, study design, baselines and comparators, outcomes and reported barriers. Due to the anticipated heterogeneity of study designs and reported outcomes a narrative synthesis is presented.

## Results

**Figure 1:**
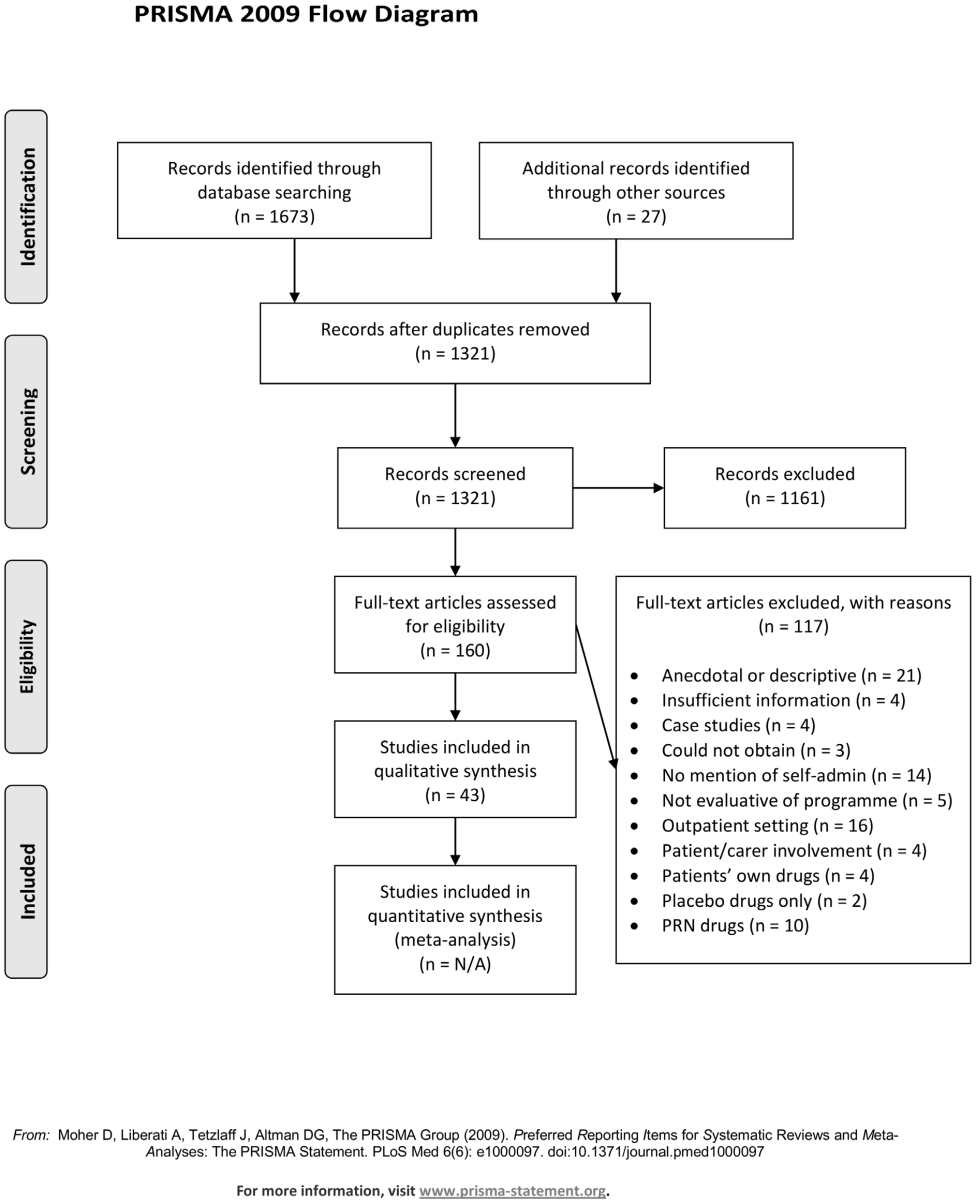
PRISMA flow diagram of study selection.

The searches identified 1321 articles, and from these 160 studies were independently selected by two reviewers based on titles and abstracts (see Figure 1). One hundred and seventeen studies were excluded. The most common reasons for excluding studies were that they did not carry out an intervention (n = 31), were anecdotal or descriptive only (n = 21), or took place in an outpatient setting (n = 16). We were unable to obtain three articles through online, local or national resources. Regarding the inclusion or exclusion of articles through to the full text screening, inter-rater reliability for the four pairs of reviewers (SR and HB; SR and GB; SR and JC; HB and GB) ranged between 0.45 (95% confidence interval 0.30 to 0.60) to 0.83 (95% confidence interval 0.72 to 0.94). These scores represent fair to excellent agreement between reviewers [18]. A total of 43 studies were included in this review.

### Study design

The characteristics of the 43 included studies are shown in Table S2. All of the studies included in this review originated in English-speaking countries and were published in the English language. Countries included the United Kingdom (n = 18), the United States (n = 15), Canada (n = 5), Australia (n = 3), New Zealand (n = 1) and Ireland (n = 1). A variety of study designs were used: case series (n = 11); before-and-after (n = 8); non-randomised controlled trial (n = 8); randomised Controlled trials (RCT; n = 6); cross-sectional (n = 5), prospective cohort (n = 4) and cohort (n = 1). In terms of the quality of study design and reporting and as can be seen in Table S2, 20 studies [9], [10], [19]–[36] scored ≧15 out of 28 on the modified version of the Downs and Black quality assessment scale [14]. We have emphasised our discussion around studies which achieved the highest quality scores. In general, higher quality assessment scores were found for RCTs (median: 16.5, range: 14–20) than for other studies (median: 12, range: 4–20).

### Structure of the SAM schemes

The rationale behind implementing a SAM scheme in each study was either explicitly stated or it was possible to infer the rationale from the article. The most common reasons for implementing SAM schemes were to improve patient compliance (n = 28), to improve patient knowledge (n = 18), to increase patient independence (n = 14) and to reduce medication errors (n = 4); most studies provided multiple reasons.

Of the 43 interventions, 10 SAM schemes had a single-staged approach to patient self-administration [21], [22], [25], [31], [36]–[41], two had two stages [24], [42], 12 had three stages [9], [10], [20], [26], [27], [30], [32], [34], [43]–[46], four had four stages[19], [47]–[49], two had five stages [28], [33], one had six stages [35], one had seven stages [50] and one paper described an intervention comprising of nine stages of increasing patient independence [51]. For the remaining ten studies, it was not clear how many stages were used [23], [29], [52]–[59]. Most studies (n = 40) had an educational element to their SAM scheme. For the remaining three studies, there was not enough information to determine whether an educational element existed [23], [52], [57]. In the majority of papers (n = 20) the educator was a pharmacist [20]–[22], [27]–[30], [32], [34]–[36], [38]–[40], [44], [46], [48], [50], [54], [56], [58], in seven a nurse [9], [19], [25], [41], [47], [51], [59] and in six both a nurse and pharmacist [10], [26], [42], [43], [49], [55]. Two studies relied solely on education through written material [33], [53], in two studies it was not possible to determine who delivered the educational element [24], [37], in one study education was provided by a physician [31], and in one study education was provided by a psychologist [45].

The types of drugs self-administered varied between studies, often reflecting different study populations. Most studies did not specify which drugs were self-administered (n = 23) or simply allowed all drugs to be self-administered (n = 3). Commonly described self-administered drugs included psychotropic medicines [38], [45], [50], insulin [23], [34] and regular oral medication [34], [56]. Drugs commonly excluded from SAM schemes were parenteral medication [10], [20], [28], [29], [31], [39], [49], drugs of dependence [10], [19], [20], [36], [51], insulin [10], [29], [31], as required (PRN) drugs [19], [36], [49], antibiotics [36], [49], and controlled substances [49], [58]. Drugs included in some interventions (e.g. insulin) were excluded in other interventions.

### Evaluation of SAM schemes

Outcome measures were assessed at a variety of time points across the 43 studies, from admission to a maximum of 24 months after patients started the SAM scheme [45]. In those studies in which patients were followed up after discharge, the median follow-up period was 40 days post-discharge (range: 0–730 days), though not all follow-up periods were explicitly specified. Multiple outcomes were measured in 26 of the 42 studies. The most common outcome measures used for evaluating SAM schemes were ‘patient knowledge’ and ‘patient satisfaction’, with 19 studies investigating each of these. Other common outcomes included compliance (n = 16) and patient success in self-administering (n = 12). Below is a summary of the findings regarding each outcome and more detailed results can be found in Table S3.

#### Patient knowledge

Nineteen studies assessed patients' knowledge of their own medicines [9], [19], [20], [24], [27], [28], [32]–[35], [37], [38], [40], [41], [43], [44], [49], [53], [59]. Knowledge topics assessed included drug name, purpose, appearance, dosage, frequency (or time of administration), and side-effects. Variation was found in the way that knowledge about medicines was both measured and reported. Eight of the 19 studies reported some significant improvement in knowledge which could be attributed to participation in SAM schemes [20], [24], [26], [27], [33], [35], [44], [53]. Two studies found that self-administering their own medicines was associated with a decrease in patients' medicines knowledge in one [28] or two [19] knowledge areas. Knowledge of side-effects was the topic that patients knew least about both before and after intervention (n = 11; n = 10 studies, respectively).

Of the five RCTs that measured patient knowledge, four found a greater increase in knowledge in SAM participants compared with control participants [27], [33], [34], [41]. However when different subsections of knowledge were individually analysed both Proos and colleagues [33] (quality score of 17) and Tan [34] (quality score of 16) found that there were no group differences regarding knowledge of medication dosage. One RCT [32] found no group differences in the degree to which knowledge increased. Two non-RCTs achieved quality assessment scores which were comparable to the RCT scores for study quality. Jensen [24] (quality score of 20) found significantly higher knowledge scores for self-administering patients compared with controls both in hospital and at discharge, while Trapp and colleagues [28] (quality score of 19) found a significant difference between groups at discharge but no significant differences at 6-weeks follow-up; however no post-hoc tests were completed and so it was not possible to understand the nature of this difference.

#### Patient compliance/error

Twenty four studies measured patient compliance/error [10], [20]–[33], [39]–[41], [47], [49], [52], [54], [58], [59]. The terminology used (e.g. medication error vs. compliance) varied between studies however the outcome measures were often identical; therefore all of these studies are discussed together.Common methods for measuring this outcome included pill counts (or a comparable alternative) conducted by staff (n = 13) [20], [22], [23], [27]–[30], [32], [33], [39], [41], [47], [54] and patient self-reported compliance (n = 10) [10], [21], [24], [26], [31], [40], [41], [49], [52], [59]. One study measured patient compliance/error through patient urine samples [25] and one study measured compliance/error through disguised observation [58]. Of the studies, nine reported greater compliance or fewer errors in SAM groups compared with control groups [20], [21], [23], [24], [27], [30], [32], [33], [52]; in five studies this difference was statistically significant [20], [23], [27], [30], [32]. Two of these five studies gained the equal highest quality assessment score for those studies measuring patient compliance. Three studies found lower compliance or greater error in SAM scheme participants compared with controls [25], [40], [58] and another study found no differences in a SAM group compared with controls [41]. Wandless and Davie [29] found that patients who received standard verbal instructions (i.e. the purpose of the trial was explained and the name, purpose, dose, and time of administration of each drug were described to the patient) and a calendar aid as part of the SAM scheme made significantly more medication errors than those patients who received only standard verbal instructions (p<0.0005) and a medication card (p<0.005). A third group receiving verbal instructions only made significantly more errors than both of the other groups (p<0.005 and p<0.01, respectively). A further two studies found no medication errors attributable to the SAM scheme over the course of their respective study periods [10], [49].

Four studies compared the change in compliance over time in patients who were self-administering [22], [26], [30], [32]. Lam and colleagues [26], whose study received a quality score of 17, found a significant reduction in non-compliant behaviours (e.g. varying the recommended medication management) and a non-significant increase in compliant behaviours (e.g. having strict routines for regular medication use). Seven studies were descriptive and reported percentage compliance to medication regimens ranging from 40% to 100% [28], [47], [54], [59] or error rates of between 2.5% and 7.5% in self-administering patients [31], [39], [58]. Of these, Trapp and colleagues' study [28], which reported 100% compliance, received the highest quality assessment score of 19.

#### Patient satisfaction

Nineteen studies investigated patient satisfaction with SAM programmes [10], [20], [23]–[25], [27], [31], [32], [34], [37], [39], [42], [43], [49], [51]–[53], [57], [59]. Patient satisfaction was measured either via a questionnaire (n = 16) or an interview (n3). However, questionnaire design was not consistent across studies. The majority of studies reported positive responses from patients regarding the SAM schemes. In several studies participants reported overall satisfaction levels of 90% to 100% [10], [24], [25], [42], [59] or recommended that the SAM scheme be continued following its evaluation [31]. Furthermore, in a study of diabetic patients whose medications were nurse-administered 71% would prefer to have self-administered their insulin [23]. The reasons patients would prefer to self-administer included increased independence [51], [59], control [23], [27], confidence [24], [27], [43] and knowledge [24], [27], [43], [51]. However not all responses were positive. In one study [34] 19% of the 14 participants were dissatisfied with the programme (measured on a 5-point Likert scale). In two studies, [52], [57] patients involved in SAM schemes reported greater satisfaction with discharge than control groups, but were not more satisfied with the medication information they received. Of the three RCTs [27], [32], [34] and highest quality non-RCT [24], there were mixed findings regarding patient satisfaction with SAM schemes.

#### Patient success/programme completion

Success within the SAM scheme was measured in 12 studies [10], [26], [32], [36], [37], [42], [45], [46], [48], [50], [55], [56]. In eleven studies (all excluding DeProspero & Wiffle [45]), the number or percentage of participants successfully participating in and/or completing the SAM scheme was recorded. This value ranged from 26% [56] to 86% [55], although what constituted success/failure differed between studies. In three studies, it was found that the percentage of participants successfully self-administering decreased after discharge [32], [37], [55]. Patients sometimes required additional intervention, such as simplified drug regimens or memory aids, to ensure their success (e.g. Fuller [37]). DeProspero and Wiffle [45] found that those patients who were successful had a shorter length of stay and fewer readmissions, compared with unsuccessful patients. Factors affecting patients' failure to complete SAM schemes included memory impairment, additional medical problems, and insufficient time spent in hospital [48], as well as increasing age and lower scores on the mini mental state examination (MMSE) [36]. The highest quality studies [26], [32] offered contradictory findings; Lam and colleagues [26] (a before-and after study) found that competency to self-administer increased significantly from admission to discharge in those patients involved in a SAM scheme, while Pereles and colleagues [32] found that participation was not a significant predictor of successful self-administration following discharge.

#### Staff satisfaction

Seven studies investigated staff satisfaction with SAM schemes [10], [19], [24], [31], [42], [49], [52]. Of these, six studies used questionnaires to measure staff satisfaction [10], [19], [24], [42], [49], [52] but none used identical questions or scales. The method used to obtain results could not be determined in the final study [31]. In general, staff satisfaction regarding SAM schemes was high. For example, Desborough and Wiffle [52] found that 91% of staff would prefer to use SAM schemes. Other staff–reported benefits of self-administration included increased structured teaching and participation by patients alongside increased follow-up by members of the multidisciplinary team [49]. Some staff reported an increased workload following the implementation of the SAM scheme [10], but these same staff also highlighted benefits of increasing nurses' medicines knowledge and satisfaction with collaboration between nurses, pharmacists, patients, and their carers. Jensen and colleagues [24] reported that nearly a quarter (23%) of nurses believed medication errors had increased due to the SAM scheme. Furthermore, staff believed the programmes to be time consuming and increase work stress, although these disadvantages were eliminated once the patient was following their protocol reliably [24]. Nonetheless, 75% of staff in Jensen's non-RCT [24] (which gained the highest quality assessment score of the studies measuring staff satisfaction) agreed the programme was beneficial, although only 34% would choose to use SAM schemes rather than nurse administration of medications.

#### Workload

Seven studies investigated the effect of SAM schemes on staff workload [10], [24], [31], [48], [52], [56], [58]. Four studies noted increased time spent on preparing medications and medication cards, as well as other clerical work [10], [48], [56], [58]. However one study noted that such tasks were eliminated [31]. Additional time was also spent educating patients about their medications. The increased workload was reported to take pharmacists between an additional 3 hours per week [56] to 30 minutes per day [48]. Two higher quality studies found that nurses observed reductions in time spent on both medication administration and discharge [52] and that the time spent on prescriptions and dispensing upon patient discharge was eliminated [10]. Anecdotal evidence from Pearce's [56] study also suggested that the scheme saved nurses time on their drug rounds, while 22% of nurses in Jensen's [24] study reported that the SAM scheme saved them time once patients reliably conformed to the SAM scheme protocol. However, 63% reported that the scheme was time consuming and 24% felt that it added work and/or stress. Traiger and Bui [49] also stated that clinical nurse specialist and pharmacy staff spent approximately 7.5 hours teaching and dispensing medications, but it was unclear how this was measured.

#### Costs

Financial costs of implementation were not explicitly measured by any of the studies reviewed. However, anecdotal evidence from three studies suggested that SAM schemes may increase costs due to supplying individual patient medicines and memory aids [42], providing lockable medicine boxes [37], and costs of materials and personnel time [49]. Four studies suggested that self-administration may improve outpatient compliance and that this may reduce re-admissions and further treatment, therefore reducing costs [27], [35], [39], [43].

## Discussion

In the current review we observed mixed findings concerning the effectiveness of implementing SAM schemes on a variety of patient, staff and institution-related outcomes. We were able to answer the following research questions:

### What types of SAM scheme interventions have been implemented among hospital inpatients?

Types of interventions varied broadly in their specific structures (e.g. numbers of stages within a SAM scheme), but in principle were very similar in allowing patients to self-administer with increasing independence and decreasing staff involvement. This was reflected in the consistency of authors' rationales for implementing SAM schemes, which were comparable across all studies. A variety of drugs were self-administered in the included studies, and a number of different patient groups assessed. It is clear from the patient populations utilised in these studies that chronic illness is a focus for self-administration programmes.

### What effects have these interventions had on outcomes related to patients, staff and on the institution?

Overall the effect of SAM scheme interventions appears mixed. Some studies concluded that SAM schemes had positive effects on the various outcome measures whilst others provided neutral or inconclusive, though rarely negative, conclusions. There was some evidence to suggest that patients' medicines knowledge increases through involvement in a SAM scheme, but not whether this involvement improves compliance, as has been suggested in some research [27], [59]. Similarly, there was little evidence of reduced medication errors. Both patients and staff responded positively when asked to rate their satisfaction with inpatient SAM schemes, yet not all staff were willing to participate in future SAM schemes. This may reflect the current culture in hospitals, where there is an expectation from patients that they will assume a more passive role and an expectation from the staff that they will assume responsibility for patients' medical care, regardless of patients' level of involvement in their care prior to admission. Alternatively, it may be a response to the initial increases in some aspects of staff workload, such as patient education and preparation of the relevant SAM scheme packages [56]. However, such increases may be coupled with a reduction in time spent on preparing and dispensing medications at discharge [10]. Furthermore, in this review we excluded studies in which carers or relatives were involved in the administration of patients' medicines. In reality, external support from carers or relatives is desirable due to the positive impact that a familiar carer may have on patients' willingness to participate and represents a significant cost saving [60], alongside the potential for reducing staff workload if relatives are willing to be involved in patients' care.

Most patients were successful in self-administering medications, and it is possible that certain patient, setting or medication factors are related to the success of SAM schemes. We noted some weak evidence that those patients with greater cognitive function were more likely to be successful [36], [48]; however these patients may have been targeted because they were more likely to successfully self-administer [26], [40], and due to the heteregenous nature of the studies it is not possible to determine the extent to which this relationship may exist. In some cases patients failed to progress through all stages of the SAM scheme or had to withdraw, which may suggest that SAM schemes are not universally appropriate for all patients in all medical specialties. However, that these patients were identified also suggests that SAM schemes may be an effective way to identify patients who would fail at self-administration at home after discharge, which may prevent potential adverse outcomes for these patients. In some studies this identification allowed for interventions to be implemented, such as altering patients' prescriptions [37] or the use of a dose administration aid [36].

There are a number of potential barriers to the implementation of and success within SAM schemes, such as the need for additional pharmacist time in preparing medicines [56] and difficulties for patients in, for example, physically opening containers [9], [27], [28], [36], [37], [41], [46]–[48], [54], [55], reading the medication list [27], [29], [30], [36], [37], [41], [46], requesting, sorting, or administering medications [36], and comprehending the importance of managing the medication regimen correctly [10], [28], [30], [33], as well as some negative attitudes towards self-administration [61]. Nonetheless, there are also factors which may facilitate success, such as patients' desire for independence [20], [42] and simplifying medication regimens [27] or instructions [46], or providing an administration aid [36]. These factors should be considered when establishing future SAM schemes.

It may be challenging to draw extensive conclusions from the included studies as many lack methodological rigour, as reflected by the wide variation in the quality assessment scores. Similar to the previously conducted literature reviews in this area [11], [12], one major issue with many of the studies in our review was that most measures had been specifically designed for individual studies in relation to their individual SAM schemes and had not been validated. The variation between the measures used in different studies made it difficult to compare results across studies and suggests that a greater consistency within studies of identified patient groups or settings (e.g. elderly care, polypharmacy, intermediate care) is required to identify generalizable factors affecting outcomes (e.g. compliance with the medication regimen; knowledge of medicines; confidence in self-medicating) of SAM schemes. The use of validated measures [62], [63] would be the ideal solution. Akin to this, there were many instances when different authors considered a single outcome measure (e.g. pill count) to be measuring difference concepts (e.g. medication errors or compliance) This suggests a need to establish not only standardised outcome measures but also standardised terminology and reporting classification systems, particularly in relation to medication error and compliance, for which the exact definitions are currently unclear [64]–[66]. Furthermore, in many studies the results were not clearly described or there was a lack of inferential statistical analysis. Finally, van Campen *et al* argue that patient satisfaction scores are often misrepresented [67], meaning that it might not be possible to draw extensive conclusions from the included studies' findings with regard to this outcome. The recent surge in the use of IT systems in hospitals may help to provide an environment in which accurate and standardised measuring and reporting can be achieved. In addition, many of the studies used small sample sizes and only eighteen out of 42 had a follow-up period beyond patients' discharge from hospital.

### Limitations

Our study has a number of limitations. Despite performing a rigorous literature search, almost half (19 out of 42) of our final studies were identified through hand-searching after our electronic searches were complete. This may suggest that our original search was not broad enough to capture all relevant papers in the first instance, or that the articles found by hand searching were not well indexed. Although our search was not restricted by language, some language bias was still inherent as our search was conducted using English-language search terms. Only two papers that were not written in the English language were assessed at full-text level; both were eventually excluded as they did not fit our inclusion criteria. It may be possible that we have excluded alternative cultural perspectives on self-administration or that the practice of self-administration itself is more prevalent in English-speaking countries. Finally, a strength of our review is that we assessed the included studies for methodological quality; despite using an adapted version of the Downs and Black quality assessment scale we still believe the adapted version provides a good indication of the relative quality of studies both within and outside of this review [15].

## Conclusions

Overall, some significant improvements in patient knowledge and compliance due to SAM schemes were observed; however these findings were not consistent across studies. There were varied findings regarding patient and staff satisfaction with SAM schemes and the success of patients within such schemes. A number of barriers to implementing SAM schemes were identified, including increased staff workload, costs, and physical difficulties for patients. It appears that, in some situations or groups, SAM schemes may be effective in helping patients to increase their independence and regain control over some aspects of their care through improved knowledge of and greater compliance with their medication regimen. An increased sense of control may be particularly poignant for specific patient groups for whom hospital spells may be long-lasting or frequent, such as those managing chronic conditions. Further research of good methodological quality and the development of standardised measures are required to successfully evaluate the effect of SAM schemes on a variety of patient, staff and system outcomes. It is important to ensure that SAM schemes balance the benefits of patient independence and increasing knowledge with the potential for increased demands on staff time and the financial implications of implementation.

## Supporting Information

Table S1 **Database search strategies.**
(DOCX)

Table S2 **Study characteristics by design.**
(DOCX)

Table S3 **Study outcomes and results by design.**
(DOCX)

Checklist S1 **PRISMA 2009 Checklist.**
(DOC)
