# Supplementary material for: Evaluating the Effectiveness of Self-Administration of Medication (SAM) Schemes in the Hospital Setting: A Systematic Review of the Literature
Source: PLoS One. 2014 Dec 2;9(12):e113912. doi: 10.1371/journal.pone.0113912 (PMC4252074; doi:10.1371/journal.pone.0113912)
Supplement: Table S1 — Database search strategies. (DOCX) [file pone.0113912.s001.docx]

**Table S1: Database search strategies**

| Database | Period | Search terms |
| --- | --- | --- |
| EMBASE via OVID | 1980–2013 | 1. self admin$.mp. 2. self administration.mp. 3. drug self administration^#^ 4. OR/1-3 5. hospital$.mp. 6. hospital/ 7. inpatient$.mp. 8. hospital patient/ 9. OR/5-9 10. adherence.mp. 11. compliance.mp. 12. error.mp. 13. error/ 14. medication error/ 15. patient satisfaction.mp. 16. patient satisfaction/ 17. knowledge.mp. 18. knowledge/ 19. medication.mp. 20. self efficacy.mp. 21. recovery.mp. 22. OR/10-21 23. drug.mp. 24. drug therapy/ 25. prescription drug/ 26. medication.mp. 27. OR/23-26 28. 4 AND 9 AND 22 AND 27 |
| MEDLINE via OVID | 1950­–2013 | 1. Self admin$.mp. 2. Self administration.mp. 3. Self Administration/ 4. OR/1-3 5. hospital$.mp. 6. Hospitals/ 7. inpatient$.mp. 8. Inpatients/ 9. OR/5-8 10. Adherence.mp. 11. Medication Adherence/ 12. compliance.mp. 13. Compliance/ 14. Medication Compliance/ 15. error.mp. 16. Medication Errors/ 17. patient satisfaction.mp. 18. Patient Satisfaction/ 19. Knowledge.mp. 20. Knowledge/ 21. Patient Medication Knowledge/ 22. self efficacy.mp. 23. Self Efficacy/ 24. Recovery.mp. 25. OR/10-24 26. Drug.mp. 27. Pharmaceutical Preparations/ 28. Medication.mp. 29. OR/26-28 30. 4 AND 9 AND 25 AND 29 |
| CINAHL Plus via OVID | 1981–2013 | 1. Self admin$ 2. “Self administration” 3. OR/1-2 4. hospital$ 5. “hospital” 6. inpatient$ 7. “Inpatients” 8. OR/4-7 9. adherence 10. compliance 11. “Medication Compliance” 12. error 13. “Medication Errors” 14. patient satisfaction 15. “Patient Satisfaction” 16. knowledge 17. “Knowledge” 18. self efficacy 19. “Self Efficacy” 20. recovery 21. “Recovery” 22. OR/9-21 23. drug 24. medication 25. OR/23-24 26. 3 AND 8 AND 22 AND 25 |
| PsycINFO via OVID | 1967–2013 | 1. exp Drug Self Administration/ 2. self administration.mp. 3. exp Drug Administration Methods/ 4. OR/1-3 5. exp Hospitals/ 6. exp Hospital$.mp. 7. exp Hospitalized Patients/ 8. exp inpatient$.mp. 9. OR/5-8 10. adherence 11. Compliance/ 12. exp compliance 13. exp Treatment Compliance/ 14. error.mp. 15. patient satisfaction.mp. 16. exp Client Satisfaction/ 17. knowledge.mp. 18. exp Health Knowledge/ 19. self efficacy.mp. 20. exp Self Efficacy/ 21. recovery.mp. 22. exp Recovery (Disorders)/ 23. OR/10-22 24. drug.mp. 25. exp Prescription Drugs/ 26. medication.mp. 27. exp Drug Therapy/ 28. OR/24-27 29. 4 AND 9 AND 23 AND 28 |
| HMIC via OVID | 1979–2013 | 1. self admin$.mp. 2. self administration.mp. 3. exp Drug administration/ 4. exp Self medication/ 5. OR/1-4 6. hospital.mp. 7. exp hospitals/ 8. inpatient.mp. 9. in patients/ 10. hospital patients/ 11. OR/6-10 12. Adherence.mp. 13. Compliance.mp. 14. exp Patient compliance/ 15. exp Drug compliance/ 16. error.mp. 17. exp Errors/ 18. exp Medication errors/ 19. patient satisfaction.mp. 20. exp patient satisfaction/ 21. knowledge.mp. 22. exp Knowledge/ 23. exp Patient knowledge/ 24. self efficacy.mp. 25. recovery.mp. 26. exp Patient recovery/ 27. OR/12-26 28. drug.mp. 29. exp Prescription drugs/ 30. Medication.mp. 31. exp Drug administration/ 32. OR/28-31 33. 5 AND 11 AND 27 AND 32 |
| NELM (previously PharmLine) | 1978–2013 | 1. “hospital self administration” |
| CPCI-S and CPCI- SSH via Web of Knowledge | 1990–2013 | 1. “hospital self administration” |
| Zetoc | 1993–2013 | 1. “hospital self administration” |
| Clinical Trials Register |  | 1. hospital.ti 2. “self administration”.ti |
| World Health Organization’s Clinical Trials Registry Platform |  | 1. “hospital self administration”.ti |
| NIHR HTA (National Institute for Health Research Health Technology Assessment) |  | 1. hospital self administration.ti |

/ = MESH terms

$ = truncation symbol used in search to find all endings (e.g. self admin$ for self administration, self administered etc.)
