# Supplementary material for: Evaluating the Effectiveness of Self-Administration of Medication (SAM) Schemes in the Hospital Setting: A Systematic Review of the Literature
Source: PLoS One. 2014 Dec 2;9(12):e113912. doi: 10.1371/journal.pone.0113912 (PMC4252074; doi:10.1371/journal.pone.0113912)
Supplement: Table S2 — Study characteristics by design. (DOCX) [file pone.0113912.s002.docx]

**Table S2: Study characteristics by design**

| **Author** | **Country** | **Study Design** | **Population** | **Purpose of SAM scheme** | **Number of stages** | **Sample Size** | **Quality Score** |
| --- | --- | --- | --- | --- | --- | --- | --- |
| Barry (1993) | USA | Before-and-after | Renal transplant patients | To improve post-discharge adherence and medicines knowledge | 4 | 99 (20 Pre-test; 79 post-test)* | 15 |
| Desborough (2009) | UK | Before-and-after | Whole hospital population | To empower patients, improve concordance, adherence and satisfaction | Not explicitly stated | 59 (32 SAM; 27 control)^$^ | 11 |
| Fuller (1995) | UK | Before-and-after | Elderly patients | To improve the care and rehabilitation offered to patients | 1 | 36 | 5 |
| Hoffman (1978) | USA | Before-and-after | Obstetric patients | To encourage independence and self-confidence in a convenient way | Not explicitly stated | 58 | 10 |
| Lam (2011) | Australia | Before-and-after | One Rehabilitation unit | To reduce medication errors and improve patient adherence post-discharge | 3 | 24 | 18 |
| Noy (1997) | UK | Before-and-after | One Post-coronary care unit | To assess patient adherence | 3 | 52 (19 post-discharge) | 7 |
| Pelletier (1983) | USA | Before-and-after | Whole hospital population | To improve patient independence and knowledge | 3 | 33 | 11 |
| Thomas (1983) | USA | Before-and-after | Renal transplant patients | To educate patients and improve adherence | 6 | 20 | 15 |
| Carter (1999) | Ireland | Case series | One Nursing Development Unit | To improve adherence and medicines knowledge | 4 | 20 | 5 |
| DeProspero (1997) | USA | Case series | Whole hospital population | To improve patient compliance | 3 | 58 | 10 |
| Grantham (2006) | Australia | Case series | One general medical and surgical ward | To improve patient knowledge and assessment of knowledge | 3 | 207** | 15 |
| Hannay (1977) | Canada | Case series | One rehabilitation unit | To decrease length of stay, improve post-discharge convalescence, avoid readmission, and reduce costs | Not explicitly stated | 13 | 8 |
| Hill (1992) | UK | Case series | Elderly patients | To improve knowledge and adherence | 3 (plus 5 steps prior to SAM) | 49 | 10 |
| Lugg (1997) | UK | Case series | Psychiatric patients | To encourage patient participation in treatment plans (Prompted by Ashworth Inquiry Report, 1992) | 7 | 14 | 12 |
| Macauley (1980) | USA | Case series | One rehabilitation unit | To educate patients and develop safe practice | Not explicitly stated | 25 | 11 |
| Ng (1996) | Canada | Case series | Psychiatric patients | To improve compliance | 1 | 15 | 11 |
| Pearce (1991) | New Zealand | Case series | Cardiology patients | To increase patient responsibility; to create medicines profile for patients; to save nurses time | Not explicitly stated | 106 | 4 |
| Reibel (1969) | USA | Case series | One rehabilitation unit | To increase patient independence | 1 | 27 (25 at discharge) | 15 |
| Taylor (1984) | Canada | Case series | One rehabilitation unit | To improve patient adherence, save nurses’ time and allow pharmacist to identify drug incompatibilities pre-discharge | 4 | 46 | 13 |
| Gandopadhyay (2008) | UK | Cohort | Diabetic patients | To improve timeliness of insulin administration in hospitals | Not explicitly stated | 35 (10 SAM; 25 control) | 16 |
| Buchanan (1972) | USA | Cross sectional | Cardiology patients | To improve compliance, to increase patient responsibility and self confidence. To increase convenience for patients and staff in hospital | 1 | 26 | 12 |
| Burrell (1998) | UK | Cross sectional | One rehabilitation unit | To improve patient knowledge, confidence, independence, self-esteem, compliance, and overcome challenges (e.g. opening bottles) | 2 | 59^$$^ | 6 |
| Deeks (2000) | UK | Cross sectional | Two acute medical wards | To improve patient knowledge, adherence and independence | Not explicitly stated | 152 | 11 |
| Kallas (1984) | USA | Cross-sectional | One rheumatic disease unit | To improve patient independence and adherence | 9 | 19 | 4 |
| Traiger (1997) | UK | Cross-sectional | Cardiothoracic transplant patients | To educate patients and improve adherence | 4 | 10*** | 10 |
| Beardsley (1982) | USA | Non-RCT | Whole hospital population | To improve health through post-discharge compliance | 3 | 64 (34 SAM; 30 control) | 15 |
| Bream (1985) | UK | Non-RCT | Elderly patients | To improve compliance | 3 | 41 (20 SAM; 21 control) | 17 |
| Cole (1971) | USA | Non-RCT | Whole hospital population | To improve patient knowledge | 1 | 75 (25 SAM; 25 discharge pharmacy consultation; 25 control) | 16 |
| Jensen (2003) | Canada | Non-RCT | Cardiology patients | To improve knowledge and adherence | 2 | 350  (178 SAM; 172 control) | 20 |
| Newcomer (1974) | USA | Non-RCT | Post-operative patients | To improve patient independence | 1 | 100 (50 SAM – monitored or not monitored; 50 control) | 12 |
| Roberts (1972) | USA | Non-RCT | Two nursing rehabilitation units | To educate patients and increase patient motivation | Not explicitly stated (1) | 54 (15 SAM; 37 control; 2 monitored SAM) | 10 |
| Trapp (1998) | UK | Non-RCT | Cystic fibrosis patients | To increase patient participation, knowledge, and adherence post-discharge | 5 | 38 (12 SAM; 12 education only; 14 control) | 19 |
| Wood (1992) | UK | Non-RCT | Elderly patients | To avoid non-adherence by incorrect dosage | 3 | 22 (11 SAM; 11 control) | 16 |
| Furlong (1996) | UK | Prospective cohort | One hospital ward | To promote self-care and improve patient knowledge and adherence | Not explicitly stated | 58 (50 SAM; 8 control) | 10 |
| Klein (1974) | USA | Prospective cohort | Psychiatric patients | To improve patient self-determination and adherence | 1 | 40 (SAM with/without instruction and no SAM with/without instruction – 10 per group) | 15 |
| Tran (2011) | Australia | Prospective cohort | Elderly patients | To improve patient autonomy and understanding of their medication regimen, and to identify medication management barriers | 1 | 62 | 15 |
| Wandless (1977) | UK | Prospective cohort | Elderly patients | To educate, to improve knowledge and adherence and to reduce medication errors | Not explicitly stated | 46 | 16 |
| Bird (1990) | UK | RCT | One 20-bed, mixed sex, acute medical unit | To enable patients to learn about and take responsibility for taking their own drugs (and compliance) | 1 | 28 (14 SAM; 14 control) | 14 |
| Foster (1993) | UK | RCT | Two general medical wards | To improve adherence | 1 | 46 (22 SAM; 24 control) | 16 |
| Lowe (1995) | UK | RCT | Elderly patients | To improve patient independence after education | 3 | 79 (42 self-administered; 37 nurse-administered) | 20 |
| Pereles (1996) | Canada | RCT | Elderly patients | To address non-adherence, drug interactions and adverse side-effects in older patients | 3 | 74 (37 self-administered; 37 nurse-administered) | 20 |
| Proos (1992) | USA | RCT | Two general medical and surgical wards | To improve patient knowledge and adherence | 5 | 47 (25 SAM; 22 control) | 17 |
| Tan (2006) | UK | RCT | Haematology patients | To educate patients and improve adherence (Prompted by ‘A Spoonful of Sugar’) | Not explicitly stated | 28 (14 SAM; 14 control) | 16 |

**Sample size plus additional: *20 staff members; ^$^46 staff members; **9 staff members; ^$$^staff members (number not stated); ***12 staff members**
