# Supplementary material for: Evaluating the Effectiveness of Self-Administration of Medication (SAM) Schemes in the Hospital Setting: A Systematic Review of the Literature
Source: PLoS One. 2014 Dec 2;9(12):e113912. doi: 10.1371/journal.pone.0113912 (PMC4252074; doi:10.1371/journal.pone.0113912)
Supplement: Table S3 — Study outcomes and results by design. (DOCX) [file pone.0113912.s003.docx]

**Table S3: Study outcomes and results by design**

| **Study Design** | **Author** | **Outcome(s)** | **Results** |
| --- | --- | --- | --- |
| Before-and-after | Barry (1993) | Patient knowledge | Knowledge screened 6 months prior to SAM scheme implementation and 1-2 days prior to discharge. Tool measured patients’ ability to state drug information (name of drug; dose; time; rationale; side-effects and special administration instructions). Results showed Knowledge was greater in SAM scheme participants, particularly about side-effects. |
|  |  | Staff satisfaction | Responses to questionnaires showed that nurses were 100% satisfied with the SAM scheme |
|  | Desborough (2009) | Patient satisfaction* | Measured via a questionnaire. No differences in satisfaction about medicines information post-intervention. However, SAM patients reported greater mean satisfaction scores with the discharge process (P=0.02) and more (90% versus 53%) agreed or strongly agreed they were involved with discussions about their medicines in hospital. |
|  |  | Patient compliance/error | No differences in self-reported compliance between intervention (n = 32) and control (n = 27) patients post-intervention. |
|  |  | Staff satisfaction | Assessed via questionnaire, 91% of 46 nurses would prefer to use SAM. 52% would be happy to use this system to supply PRN medicines, but opinion was divided on controlled drugs. |
|  |  | Workload | Most nurses agreed or strongly agreed that SAM reduces the time spent administering medicines (87%) and decreases discharge times (59%). Opinions did not differ between intervention and control wards. |
|  | Fuller (1995) | Patient knowledge | When asked by nursing staff, 53% of 36 patients knew what all drugs were and what for before involvement in the SAM scheme and 86% post-discharge. |
|  |  | Patient satisfaction | 78% of patients thought they were given enough info about drugs during admission, 75% thought information was helpful, 22% thought the information they had learned had changed their views about drugs they were taking. |
|  |  | SAM success | 43% were assessed as independent 2 weeks post-discharge; 16% managed simple drug regimens; 10% managed using drug charts; 33% could not self-administer independently even with aids. 53% received some help by family, friends or services. |
|  | Hoffman (1978) | Patient knowledge | Of 58 participants, medicines knowledge, assessed by a 10-item questionnaire improved significantly from admission to the SAM scheme to discharge in 5 questions. |
|  |  | Patient satisfaction | Of 30 respondents, the majority of patients liked having their medications at the bedside. All patients said that nursing staff were helpful in answering medication questions. 73% found a post-discharge pharmacy intern visit to be helpful. |
|  | Lam (2011) | SAM success* | Of 24 participants, 22 successfully completed stage 2 and 17 completed stage 3 of the 3-stage SAM scheme. Competency scores (assessed via the Drug Regimen Unassisted Grading Scale; DRUGS) at discharge improved significantly (P<0.001) compared with that before commencement of medication self-administration. |
|  |  | Patient compliance/error * | Assessed using the Tool for Adherence Behaviour Screening (TABS), participants self-reported compliance was high even prior to commencing the SAM scheme. Participants reported a significant decrease (P=0.02) in intentional and unintentional non-adherent behaviour and a trend towards improved adherent behaviour (P=0.08) after participation in the SAM scheme. For patients who achieved stage 3 of the scheme, compliance was assessed via tablet count and patients were found to be 98.7% compliant. |
|  | Noy (1997) | Patient knowledge | Assessed via a questionnaire, patients who self-medicated (n = 107) increased their knowledge of the names and side-effects of their drugs, the reasons for taking them and times of administration from pre-discharge to 6 weeks post-discharge. There was a high attrition rate (n = 19 at 6-weeks post discharge). |
|  |  | Patient satisfaction | Following self-medication, over 90% of patients were almost entirely satisfied with their drug knowledge and were confident of their ability to take their drugs safely and accurately. |
|  | Pelletier (1983) | Patient knowledge* | Patients’ (n = 33) knowledge (assessed via an informal oral test conducted by pharmacists and recorded into a standardised table) of each of their drugs’ names, strength, use, appearance and time of administration increased significantly from pre- to post-intervention (P<0.05 for all categories). There was no significant increase in knowledge about medication strength. Knowledge also increased as patients progressed through levels of SAM scheme. |
|  | Thomas (1983) | Patient knowledge* | Patients tested at four points (pre-education, post-education, pose-self-medication, and 1 month post-hospital discharge) Statistically significant difference in patient knowledge from pre-education (mean = 53.0% correct) to post-education (mean = 87.6% correct; P<0.01) and from post-education test (mean = 95.9% correct) to post-self-medication test (mean = 98.1% correct; P<0.01), but not from post-self-medication test to 1 month post-discharge. |
| Case Series | Carter (1999) | Patient compliance/error | Compliance was assessed via pill count (observed/expected*100). 40% of 20 patients achieved 100% compliance; 30% achieved scores between 10% and 50% compliance; 20% achieved 0% compliance despite prolonged education; 10% improved in compliance with further education. |
|  | DeProspero (1997) | SAM success* | The successful group (n = 29) was hospitalized an average of 48.6 days compared with 85 days for the unsuccessful group (n = 29 randomly selected unsuccessful patients; P<0.01). During the same period the successful group had a mean of 3 readmissions compared with 8 for the unsuccessful group (P<0.05). |
|  | Grantham (2006) | SAM success | Approximately 45% (n = 94) of participants remained on level one, while 26% (n = 54) achieved level two and 29% achieved level three (n = 59). |
|  |  | Patient compliance/error | Survey results of 34 participants showed that at discharge, 53% of respondents (n = 18) believed that they always remembered to take their medications, while 35% (n = 12) sometimes forgot and 12% (n = 4) often forgot. No patient-initiated errors occurred in the hospital during the study period. |
|  |  | Patient satisfaction | All patients surveyed reported satisfaction with the SAM scheme, the level of support provided by staff, the amount of information provided and with using a Medication Information Card. |
|  |  | Staff satisfaction | Nurses (n = 14) were surveyed at 1, 3, and 5 month intervals (response rates of 62%, 62% and 57%, respectively). They reported being satisfied with the self-medication process, and satisfaction did not change over time (on a likert scale of 1-4, mean responses were 3.53, 3.3 and 3.63 at each respective interval). |
|  |  | Workload | At 3 months post-SAM scheme implementation, 57% staff believed that the SAM scheme increased their workload and time spent on education activities, but decreased time spent on procedures related to patient discharge. |
|  | Hannay (1977) | Patient compliance/error | Compliance was measured via pill count. Patients showed an average compliance of 83% (n = 48) to prescribed self-administration schedules. All errors were omissions (i.e. non-compliance), but in some cases this was legitimate omission (e.g. preoperative patients). |
|  | Hill (1992) | SAM success | 79% of 37 female patients and 50% of 12 male patients administered and looked after their own medications successfully. |
|  | Lugg (1997) | SAM success | Of 14 psychiatric patients based at a high security hospital, the mean time for patients to progress through stages 1–7 was 7 months (range 2–10 months). One patient discontinued (no reason given) and several patients were temporarily discontinued for various reasons. |
|  | Macauley (1980) | SAM success | Twenty nine patients were recruited and 25 successfully completed the SAM scheme (of those patients who were unsuccessful, 2 patients died and 2 were discontinued). The remaining patients were discharged home (excluding one who was transferred to a chronic care facility). 22-months post-admission, 21 of the 25 successful patients who completed the SAM scheme reported to the hospital that they were managing satisfactorily at home. |
|  | Ng (1996) | Patient knowledge* | Significant improvement increase in knowledge from pre-test (mean = 45.5% questions correct) to post-test (mean = 66.9% questions correct) scores (P = 0.001).. Patients improved knowledge about specific drugs (neuroleptics, anticholinergics, and anxiolytics). Significant improvements were observed from pre-test to post-test in knowledge of side-effects (from 11.2% to 41.7%; P=0.001) and compliance concerns (54.5% to 91.9%; P=0.001). |
|  | Pearce (1991) | SAM success | 26% of patients admitted to the ward self-medicated (n = 106). This represented, on average, 8 new patients per week. |
|  |  | Workload | Data from 25 consecutive patients was measured to estimate the extra workload involved in the SAM scheme. The total extra pharmacy workload in administering the self-medication scheme was estimated at 3 hours per week. Approximately 1.5 hours of this was extra pharmacist time spent on the ward. |
|  | Reibel (1969) | Patient compliance/error | Of 1,715 total drug units administered, patients reported that 109 units were omitted and 19 units were taken incorrectly. 13 of the 27 patients committed no errors. Medication errors committed by 7 patients accounted for 87% of all errors; these patients committed errors more than 10% of the time. |
|  |  | Patient satisfaction | Upon answering a questionnaire at discharge, all patients recommended that the programme be continued. Most liked the programme because their medication was immediately available to them. |
|  |  | Staff satisfaction | All staff (n = 7) recommended that the SAM scheme be continued. They felt there was high correlation between participating in SAM and participating in other prescribed activities |
|  |  | Staff workload | The workload for physicians and nurses decreased (checking and writing medicine cards and reordering drugs were eliminated). The workload for pharmacists increased due to the automatic refilling process. |
|  | Taylor (1984) | SAM success | 36 cerebral vascular accident patients (CVA) and 10 non-CVA patients were recruited. Of the CVA patients, 94% completed stage 2; 55% completed stage 3 & 4. Of the non-CVA patients, 100% completed stage 1; 70% completed stages 3 & 4. Major limiting factors in failure to complete stage 3 were: a) memory impairment; b) development of additional medical problems; c) insufficient time to complete all stages before discharge. |
|  |  | Workload | The amount of time required for each stage varied considerably. Pharmacist spent on average 30 minutes daily on the rehab unit (method of measurement not explicitly stated). |
| Cohort | Gangopadhyay (2008) | Patient compliance/error | Compliance was measured via checks by hospital staff that insulin was administered at an appropriate time in 35 diabetic patients (10 in SAM group, 25 in control group). When insulin was self-administered, it was appropriately timed in 78% of cases, significantly better (P<0.001) than when administered by hospital staff (19% correctly timed). |
|  |  | Patient satisfaction | 15 of 21 respondents from group 1 (control group) would have preferred to self-administer insulin. The vast majority reported feeling a lack of control when they could not self-administer. |
| Cross-sectional | Buchanan (1972) | Patient satisfaction | Measured via two different questionnaires. 76% patients of 26 patients who responded preferred self-administering their medications. |
|  |  | Patient compliance/error | Measured via pill count. Out of 2,806 doses, 69 medication errors were counted (error rate 2.5%). Only 20 of 78 (26%) patients made errors. When asked as part of the questionnaire, 31% of the 26 respondents admitted to making errors. |
|  | Burrell (1998) | Patient satisfaction | Of 59 patients audited, 90% felt happier about taking their own medications. 41% felt their understanding of their medications had improved. |
|  |  | SAM success | 82% of patients admitted during the study period administered their own medicines. 18% chose not to participate or were excluded. 23% did not commence SAM within 2 days of admission. |
|  |  | Staff satisfaction | Nurses, pharmacists and clinical assistants all believed the programme benefited patients. At re-audit every nurse except one preferred the SAM scheme to the traditional 'drug trolley' round. |
|  | Deeks (2000) | Patient satisfaction* | SAM patients (n = 47) were significantly more likely than non-SAM patients (n = 100) from the same wards to report their overall care as excellent (measured on a Likert-type scale ranging from “excellent” to “dreadful”), and reported a significantly better overall impression of care (P<0.05). SAM patients were also significantly more satisfied with the discharge process (P=0.008). No group differences were found in the satisfaction with the way in which information was provided to the patient. The majority of SAM patients who were involved in the SAM scheme would choose to self-administer in a future admission (80.9%; n = 47). This was not the case for control group patients (26.5%; n = 102). |
|  | Kallas (1984) | Patient satisfaction | Feedback obtained through informal discussion and an evaluative questionnaire. 16 of 19 patients found the programme helpful. Patients commented that they enjoyed learning about medications and increasing their independence/ self-reliance. 9 felt the SAM scheme helped to solve problems/concerns and would continue at home. Only 1 problem was reported at home (confusion about dosage). |
|  | Traiger (1997) | Patient knowledge | 80% of patients could verbalize all required information at time of discharge. SAM patients knew more about their medications than the comparison group of previous transplant recipients. |
|  |  | Patient compliance/error | Method for measuring errors not stated, but no medication errors were made that were attributable to the SAM scheme. In a post-discharge survey, 2 out of 9 participants reported that they forgot to take one medication, which was not favourable compared with the control group. |
|  |  | Patient satisfaction | In a questionnaire, SAM patients reported feeling more confident about managing their medications and had less difficulty integrating the medication regimen into their daily life. |
|  |  | Staff satisfaction | Nursing staff felt the SAM scheme was an effective educational tool and worth the extra time invested by nurses and pharmacy personnel. Nurses believed SAM patients were more self-confident, more in control, and less apprehensive about post-discharge self-medicating compared with non-SAM patients. |
| Non-RCT | Beardsley (1982) | Patient compliance/error * | Measured by pill count.. Significant difference (P<0.02) in the number of days of therapy deviation between the treatment (1.8 deviation days) and control group (3.6 deviation days). |
|  |  | Patient knowledge* | Measured via questionnaires completed through interviews. Knowledge was significantly greater in the SAM group at follow up compared to entrance into the SAM scheme (P<0.01) |
|  |  | Patient satisfaction* | Patients in the SAM group were more satisfied with pharmacy services (more interaction) (P<0.01) but not hospital services (P>0.05), and felt pharmacists were more knowledgeable about prescription drugs than did patients in control group (P<0.04). |
|  | Bream (1985) | Patient knowledge | Patients assessed via an oral questionnaire administered by nurses on admission, at discharge and one month post-discharge. In SAM group all areas of knowledge (timing of administration, dose, drug use, drug name, and side effects) improved over time from admission to discharge. From admission to 1 month post-discharge, the SAM group experienced greater knowledge increase compared with the control group (not tested for significance). |
|  | Cole (1971) | Patient compliance/error | Three groups of patients (group 1: pharmacist consultation at discharge; group 2: no consultation or self-administration; group 3: consultation at discharge and self-medication). Patient compliance was measured by patient self-pill count 2 weeks after discharge. Drugs were correctly administered by 92% of patients in group 1, 76% of patients in group 2 and 88% of patients in group 3. Group 2 accounted for 84% of the errors at follow-up. Errors were most common in patients taking tranquilizers and then in patients taking antiarrhythmic agents. |
|  | Jensen (2003) | Patient knowledge* | All 350 patients increased medication knowledge (measured using the Medication Knowledge Questionnaire) 16 weeks post discharge. Medication knowledge scores in SAM group (178) were significantly higher than in nurse-administered (n = 172) in hospital (P=0.003) and at discharge (P=0.006). Medication knowledge was found to be inversely related to medication regimen complexity. |
|  |  | Patient compliance/error | There were no statistically significant differences between the SAM and nurse-administered groups on medication adherence scores. Compliance was not affected by medication regime complexity. |
|  |  | Patient satisfaction* | 73.7% of all patients would prefer to self-administer their medicines in hospital. The SAM group agreed more than the nurse-administered group that their medications were clearly explained (4.74 vs. 4.34 on a 5-point Likert scale; P=0.001), that they understood why they needed their medications (4.88 vs. 4.71; P=0.01), and their importance (4.60 vs. 4.38; P=0.01), and felt their compliance improved from admission to discharge (2.98 vs. 3.45; P=0.002). |
|  |  | Staff satisfaction | 40.9% of 44 nurses preferred nurse administration; 34% preferred SAM; 11.4% preferred a combination; 2.3% were unsure. 75% agreed SAM scheme was beneficial; 22.7% felt that medication errors had increased. |
|  |  | Workload | 63.2% of nurses reported that the SAM scheme was time-consuming and 23.7% felt that it added work/stress. |
|  | Newcomer (1974) | Patient knowledge* | Three groups were assessed (monitored self-administration; self-administration; nurse-administration). Patients who had been taught about their drugs by the pharmacist were generally more knowledgeable and were able to retain that knowledge, but there were no significant group differences. |
|  |  | Patient compliance/error | Study patients (groups 1 and 2) reported that they missed 24% of drugs at home and controls (group 3) missed 15.6% (not statistically significant). |
|  | Roberts (1972) | Patient compliance/error | Measured via disguised observation by nurses and pharmacy technicians. In the SAM group, of the 3,548 doses, 154 errors were made (error rate = 4.3%). In the monitored self-medication group, technicians made 7 errors in monitoring 63 doses (error rate = 10.1%) and nurses made 17 errors in monitoring 56 doses (error rate = 30.4%). In the nurse/technician administered group, of 1,135 doses 27 errors were committed (error rate = 2.4%). |
|  |  | Workload | Pharmacists reported that 8.7% of their time was spent on self-medication counselling. |
|  | Trapp (1998) | Patient knowledge* | Measured via questionnaire at discharge. There was a statistically significant difference in drug knowledge between groups (control mean = 2.55 out of 3; teaching group mean = 2.68; SAM group 2.68; P<0.01), although the value of this difference was not described. There was no difference at admission or at 6-week follow up. |
|  |  | Patient compliance/error | At first pill count (within 48 hours of admission) there was 100% compliance for all 8 patients in the SAM group. The average compliance at the second random pill count was 99%. |
|  | Wood (1992) | Patient compliance/error * | The SAM group (n = 18; n = 11 at 3 month follow up) were significantly more compliant than controls (n = 15; n = 11 at follow up) at 2 weeks (6% SAM group and 47% control group made >15% errors; P<0.02) and 3 months (27% SAM group and 73% control group made >15% errors; P<0.05) post-discharge, as measured via pill count. The majority of poor compliance was due to under-dosing. |
| Prospective cohort | Furlong (1996) | Patient knowledge* | All dimensions of patient knowledge, except knowledge of side-effects and precautions, had significantly improved at 8 weeks post-discharge in both groups. However, there were either no group differences or the knowledge was better in the nurse administered group in the different knowledge categories (drug name and dose; drug purpose; when drug taken; side-effects) at the different time points (on admission; on discharge; post-discharge). |
|  |  | Patient satisfaction | Ninety per cent (n = 45) of SAM group patients and) 100% (n = 8) of the nurse-administered group (n = 8) were satisfied with their medication administration while they had been in hospital. |
|  |  | Patient compliance/error | Only 2 SAM group patients reported that they had forgotten to take their medication on a single occasion while in hospital. 16 patients reported having forgotten to take their medications at least once post-discharge. |
|  | Klein (1974) | Patient compliance/error * | A higher incidence of compliance, measured via urine samples, was observed in the nurse-administered group where no instruction was provided to patients (no non-compliance was observed) compared with SAM group with instruction (20% overdose and 30% underdose), SAM group without instruction (30% overdose and 10% underdose) and nurse-administered with instruction (10% underdose). The degree of non-compliance was significantly greater in self-administering compared with non-self-administering groups (P<0.01). Instructions by nursing staff undermined rather than supported patients’ attitude toward and adherence to prescribed medications. |
|  |  | Patient satisfaction* | The Semantic Differential Survey administered to patients showed a preference for self-administration. Nurse administration was perceived as less potent (P<0.01) and less desirable (P<0.01) than self-administration by all patients over time. |
|  | Tran (2011) | Patient success* | 69.4% of 62 participants passed the SAM scheme without requiring further intervention. 11.3% passed with intervention and 19.4% failed the SAM scheme. Older patients and patients with poorer cognitive function were significantly more likely to fail (P=0.01 and P<0.01, respectively). |
|  | Wandless (1977) | Patient compliance/error* | Total error count from the 46 patients was 756. Considering all the groups (group 1: verbal instruction; group 2: calendar plus verbal instructions; group 3: standard instruction plus individual identifying card for each tablet), patients who were given an aid to memory in the form of a card (188 errors out of 1046 tablets) or a calendar (236 errors out of 1,716 tablets) made significantly fewer mistakes in their tablet taking than controls (332 errors out of 1,428 tablets; P<0.0005 and P<0.005, respectively). |
| RCT | Bird (1990) | Patient knowledge | The SAM group (n = 14) had better medicines knowledge, better side-effect knowledge, and better knowledge of illness than controls (n = 14). |
|  |  | Patient compliance/error | Compliance was measured by self-report in all patients and pill count in 9 patients per group. 100% of patients reported taking their tablets correctly, but pill count showed that compliance was better in the SAM group (7 pill counts were correct) than in controls (2 pill counts were correct). |
|  | Foster (1993) | Patient compliance/error | Compliance, measured via a 1-week post-discharge pill count, was 99% in the SAM group (n = 22) and 94% in the control group (n = 24). No statistically significant improvement in compliance was found one week after discharge in SAM patients. |
|  | Lowe (1995) | Patient knowledge* | Significantly more patients (38/42; 90%) in the SAM group knew the purpose of their medicines in the SAM group compared with those in the control group (17/37; 46%, P<0 .001). |
|  |  | Patient compliance/error * | Compliance scores, calculated as a percentage of the correct number of remaining pills on a count conducted at a 10-day post-discharge visit, were significantly higher for patients in the SAM group compared with the control group (95% vs. 83%, P<0.02). |
|  |  | Patient satisfaction | 40 patients (95% respondents) would prefer to self-administer. As a result of being involved in the SAM scheme, 37 (88%) patients felt more in control of their medicines, 18 (43%) felt more confident taking their medicines at home, and 18 (43%) said their understanding of their drug treatment had increased. |
|  | Pereles (1996) | SAM success | Participation in the SAM scheme was not a significant predictor of SAM success at discharge. |
|  |  | Patient knowledge* | From admission to discharge and follow-up, knowledge significantly increased in both groups (P<0.001). Knowledge of side-effects did not change over time. |
|  |  | Patient compliance/error * | The SAM group had significantly fewer medication errors on average at 1-month follow-up than the control group (14 vs 25). There were fewer serious errors in the SMP than control group but this difference was not significant. Controlling for age and mental status, there was a significant improvement in compliance for the SAM group (P<0.001). |
|  |  | Patient satisfaction | There was no significant difference in morale as measured by the Philadelphia Morale Scale between the SAM group and control group or over time from admission to discharge to follow-up controlling for age and sex. |
|  | Proos (1992) | Patient knowledge* | All patients in the SAM group (n = 48) and the control group (n = 44) showed significant improvements (P<0.05 for all) in all knowledge areas (medication name; frequency of administration; reason for administration; side-effects) except dosage from programme entrance to discharge. SAM patients showed greatest increase in knowledge of the reason for each medication and side-effects of the medications. |
|  |  | Patient compliance/error | Assessed via pill count at a 1-month follow-up visit. Compliance in the SAM group was 94% and in the control group was 90%. There were no statistically significant differences in compliance between groups. |
|  | Tan (2006) | Patient knowledge | Knowledge improved across all knowledge categories (assessing medication names; dosage; timing; purpose; and side-effects via a questionnaire) in all patients. The greatest improvement was in knowledge of side-effects: SAM group: mean score improved from 17.4% to 45.4%; control group: mean score improved from 19.8% to 30.5%). SAM patients (n = 14) who received education by a pharmacist had a greater medicines knowledge compared with non-SAM patients (n = 14) who received no education. |
|  |  | Patient satisfaction | 81% of all SAM group patients were very satisfied or satisfied; 19% were dissatisfied. 61% would prefer to self-administer in the future. |

^*^ Result statistically significant at alpha level of P<0.05
